# Trends in Prescriptions for Insomnia in a Province in China Between 2015 and 2019

**DOI:** 10.3389/fpsyt.2022.915823

**Published:** 2022-06-20

**Authors:** Guodong Lou, Zhenwei Yu, Liying Chen, Yiting Zhou, Lisan Zhang

**Affiliations:** ^1^Department of Pharmacy, Sir Run Run Shaw Hospital, School of Medicine, Zhejiang University, Hangzhou, China; ^2^Department of Neurology, Sir Run Run Shaw Hospital, School of Medicine, Zhejiang University, Hangzhou, China; ^3^Center for Sleep Medicine, Sir Run Run Shaw Hospital, School of Medicine, Zhejiang University, Hangzhou, China

**Keywords:** benzodiazepine, benzodiazepine receptor agonists, insomnia, pharmacological treatments, prescription

## Abstract

**Background:**

The inappropriate use of pharmacological treatments for insomnia may increase patients' risk of serious adverse events. However, few epidemiological studies on the use of medications for insomnia in China have been conducted to date.

**Objective:**

We aimed to investigate the current pharmacological treatments for insomnia and guide the rational use of drugs.

**Methods:**

The prescription data of outpatients with insomnia between 2015 and 2019 in Zhejiang province were extracted from the Hospital Prescription Analysis Cooperative Project of China and evaluated. The demographic characteristics of insomnia and the proportion and prescription trends of different drugs were analyzed along with multidrug combinations for insomnia.

**Results:**

The number of patients with insomnia who were prescribed medications for insomnia increased from 2,385 in 2015 to 3,919 in 2019, with an increase of 64.32%, whereas the mean age of these patients decreased from 64.07 years to 60.94 years. There were nearly 1.42 times as many female patients prescribed medications for insomnia as male patients, and female patients tended to be younger than male patients. Benzodiazepines (53.99%) were the most common type of medicine for insomnia. The incidence of benzodiazepine usage decreased significantly yearly (*P* < 0.01), whereas the incidences of non-benzodiazepine receptor agonist (nBZRA) and antidepressant usage increased (*P* < 0.05). The most common benzodiazepine, nBZRA, antidepressant, and antipsychotic were estazolam, zolpidem, trazodone, and olanzapine, respectively. A total of 13.97% of outpatients with insomnia were prescribed multiple drugs for insomnia, even though nearly half of the drug combinations had similar pharmacological mechanisms.

**Conclusions:**

Benzodiazepines remained the most common medication for insomnia, but the prescription rates of nBZRAs and antidepressants increased. Attention should be paid to multidrug combinations for insomnia, which may lead to an increased risk of serious adverse effects.

## Introduction

Insomnia is an extremely common sleep disorder that affects a large proportion of people worldwide. The prevalence of insomnia in the United States and Europe is 10–22 and 5–19% (1, 2), respectively, and is more commonly observed in women and older adults (2, 3). In China, ~15% of the population is diagnosed with insomnia (4), and 45% of the population reportedly experienced varying degrees of insomnia symptoms in a given month (5). Daytime dysfunctions induced by insomnia, such as fatigue, mood disorders, and cognitive impairment, affect most patients with insomnia and reduce their quality of life, as do complications such as obesity, hypertension, diabetes, and cardiovascular disease (3, 6).

Pharmacological treatments, such as benzodiazepines (BZDs), non-benzodiazepine receptor agonists (nBZRAs), antidepressants, antipsychotics, melatonin receptor agonists (MRAs), and orexin receptor antagonists (ORAs), are recommended if non-pharmacological therapies are ineffective (2, 7, 8). However, inappropriate use of these medications causes an increased risk of abuse, dependency, drug tolerance, dysmnesia, daytime sleepiness, hospitalizations, and death (9, 10). Therefore, the current pharmacological treatments for insomnia must be investigated to identify medication problems and guide the rational use of drugs.

In the United States, the prescription of BZDs (2.6–4.4%) and nBZRAs (0–1.4%) increased between 1993 and 2010, as did the use of nBZRAs (2.3–13.7%) among patients with sleep disorders, but the use of BZDs decreased (23.5–10.8%) (11). In contrast, BZD prescriptions declined and non-BZD prescriptions increased in Australia between 2011 and 2018, with a 3.2–5.9% annual increase in the proportion of prescriptions in patients recently diagnosed with insomnia (12). In Japan, the most common hypnotic drugs in patients are BZDs (59.7%), non-BZDs (36.8%), MRAs (3.1%), and ORAs (0.4%). Approximately 5.3% of the patients were prescribed a second hypnotic drug within a year after the first hypnotic drug was prescribed. Of these, 8.4% were prescribed at least three hypnotic drugs (13). BZDs are the most commonly prescribed drugs in Korea, but zolpidem is the most frequently prescribed medication for patients with insomnia who use a single sedative hypnotic drug (14).

However, few epidemiological studies have been conducted on medications for insomnia in China to date, and there is also a lack of studies on medication combinations that may increase the risk of adverse reactions in patients. Therefore, we evaluated the prescription data of patients with insomnia from the Hospital Prescription Analysis Cooperative Project in China and investigated drug trends from 2015 to 2019.

## Materials and Methods

### Ethics

This study was approved by the Ethics Committee of Sir Run Run Shaw Hospital, School of Medicine, Zhejiang University (reference number: 20210924-33). Informed consent was waived due to the retrospective nature of the study.

### Study Design

This study was designed in accordance with the statement of reporting of studies conducted using observational routinely collected health data statements for pharmacoepidemiology (RECORD-PE statement) (15).

### Data Collection and Study Population

The prescription data of outpatients with insomnia in Zhejiang province, which is one of the most economically developed provinces in China with a population of nearly 65 million, accounting for 5% of Chinese population, were extracted from the Hospital Prescription Analysis Cooperative Project of China (16). This database collects prescription information on sampling days from most general hospitals in China. The sampling form consisted of 40 random days per year, with 10 sampling days per quarter. The prescription information included prescription code, sex and age of the patient, prescription date and number, location, hospital code, clinical department, diagnosis, generic drug name, usage and dosage, total amount of medicine taken, and cost.

In the present study, outpatient prescriptions meeting the following criteria were extracted: (i) diagnosis included insomnia, anhypnia, “insomnia disorder” or “non-organic insomnia;” (ii) prescription dates fell between 2015 and 2019; (iii) all locations were in the Zhejiang province; and (iv) hospitals continuously participated in the project during the study period. Prescriptions with incomplete data were excluded.

### Drug Classification

In accordance with the Chinese guidelines (8, 17), the medications were categorized as follows: (i) BZDs (triazolam, alprazolam, lorazepam, temazepam, quazepam, flurazepam, estazolam, diazepam, clonazepam, nitrazepam, oxazepam, prazepam, and midazolam), (ii) nBZRAs (zolpidem, zaleplon, zopiclone, or dexzopiclone), (iii) antidepressants [sedative antidepressants: doxepin, trazodone, amitriptyline, and mirtazapine; non-sedative antidepressants: selective serotonin reuptake inhibitors (SSRIs), and serotonin/norepinephrine reuptake inhibitors], (iv) antipsychotics (quetiapine, olanzapine, and clozapine), (v) MRAs (agomelatine and ramelteon), (vi) ORAs (suvorexant), (vii) or “other sedative drugs” (diphenhydramine, phenobarbital, gabapentin, pregabalin, tiagabine, and chloral hydrate).

### Data Analysis

First, the demographic characteristics of patients with insomnia who had been prescribed medications for insomnia were evaluated through outpatient visits. The number of outpatient visits per year was calculated, and trends were analyzed separately according to age, sex, and clinical department using linear regression. Next, the proportions of annual and total prescriptions for different kinds or classes of drugs were calculated, and trends were analyzed using linear regression. The defined daily doses (DDDs) of each drug were calculated to evaluate the propensity of medication use. DDDs are equal to the ratio of the total doses of a certain drug to the defined daily dose value, meaning that higher DDDs equate to an increased tendency toward drug abuse (18). Lastly, combinations of different types of drugs prescribed during one outpatient visit were analyzed. The prescription data were processed using Microsoft Access software and exported to Microsoft Office Excel® 2007 (Microsoft Corp., Redmond, WA, USA) for statistical analysis. Statistically significant differences (*P* < 0.05) were determined using SPSS 22.0 (IBM Corp., Armonk, NY, USA).

## Results

### Demographic Characteristics of Patients With Insomnia

**Figure 1:**
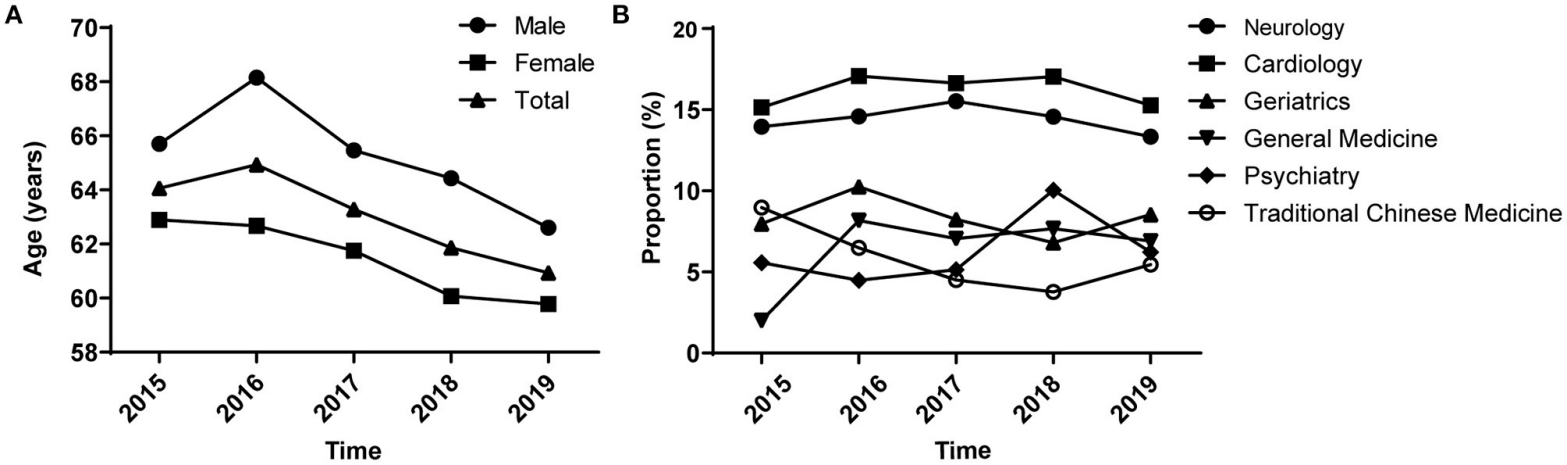
Trends of outpatients with insomnia analyzed according to sex and clinical department between 2015 and 2019. **(A)** Age trends of outpatients with insomnia by sex. **(B)** Trends by clinical department.

Data from a total of 16,133 outpatient visits by patients prescribed medications for insomnia in six general hospitals in the Zhejiang province were extracted. These six general hospitals are all famous tertiary general hospital in Zhejiang province, distributed in several major cities, whose population accounts for more than half of the Zhejiang province. Table 1 presents the demographic characteristics of the study population between 2015 and 2019. Overall, there were more female than male patients, with percentages of ~58.72 and 41.28%, respectively. The number of patients prescribed medications for insomnia increased from 2,385 in 2015 to 3,919 in 2019, equating to an increase of 64.32%. Approximately 18.07% of patients prescribed medications for insomnia were aged <45 years, 38.39% were aged 45–65 years, 24.86% were aged 66–79 years, and 18.68% were aged ≥80 years. Moreover, the percentage of patients prescribed medications for insomnia aged 45–65 and 66–79 years increased significantly between 2015 and 2019 (*P* < 0.05). The mean age of patients decreased from 64.07 years in 2015 to 60.94 years in 2019 (Figure 1). Female patients prescribed medications for insomnia in the study period tended to be younger than male patients, with the mean age of women decreasing from 62.90 years in 2015 to 59.78 years in 2019 and that of men decreasing from 65.71 years in 2015 to 62.60 years in 2019. The three most frequently visited departments for patients prescribed medications for insomnia were cardiology (16.26%), neurology (14.39%), and geriatrics (8.54%).

**Table 1:** Demographic characteristics of outpatients prescribed medications for insomnia.

	**Number of patients (%)**					
	**2015**	**2016**	**2017**	**2018**	**2019**	**Total**
Outpatient visits	2,385	3,098	3,252	3,485	3,919	16,133
Age (years)						
<45	514 (21.55)	536 (17.30)	537 (16.51)	608 (17.44)	720 (18.40)	2,915 (18.07)
45–65*	855 (35.85)	1,158 (37.38)	1,232 (37.88)	1,372 (39.37)	1,576 (40.28)	6,193 (38.39)
66–79*	557 (23.35)	759 (24.50)	824 (25.34)	875 (25.11)	996 (25.45)	4,011 (24.86)
≥80	459 (19.25)	645 (20.82)	659 (20.27)	630 (18.08)	621 (15.87)	3,014 (18.68)
Sex						
Male	979 (41.05)	1,283 (41.41)	1,333 (40.99)	1,461 (41.92)	1,603 (40.97)	6,659 (41.28)
Female	1,406 (58.95)	1,815 (58.59)	1,919 (59.01)	2,024 (58.08)	2,310 (59.03)	9,474 (58.72)

*The percentage of patients prescribed medications for insomnia increased significantly between 2015 and 2019 by linear regression (P < 0.05).

### Trends in Prescriptions by Class of Drug

The number and proportion of medical orders for the different classes of anti-insomnia drugs per year are shown in Table 2. BZDs (53.99%) were the most common type of medicine for insomnia, followed by nBZRAs (31.34%) and antidepressants (10.33%). Approximately 2.72% of the prescriptions for outpatient insomnia were for antipsychotics; only three outpatients were prescribed MRAs, and none were prescribed ORAs. The incidence of BZD usage decreased significantly every year (*P* < 0.01), whereas the incidence of nBZRA and antidepressant usage increased between 2015 and 2019 (*P* < 0.05).

**Table 2:** Trends of therapeutic drugs prescribed in sampled outpatients with insomnia between 2015 and 2019.

	**Number of prescriptions (%)**					
	**2015**	**2016**	**2017**	**2018**	**2019**	**Total**
All classes	2,730	3,605	3,817	4,056	4,548	18,756
BZDs**	1,607 (58.86)	2,047 (56.78)	2,129 (55.78)	2,091 (51.55)	2,252 (49.52)	10,126 (53.99)
nBZRAs*	757 (27.73)	1,048 (29.07)	1,102 (28.87)	1,340 (33.04)	1,632 (35.88)	5,879 (31.34)
Antidepressants#	285 (10.44)	390 (10.82)	414 (10.85)	443 (10.92)	509 (11.19)	2,041 (10.88)
Sedative antidepressants	145 (5.31)	161 (4.47)	184 (4.82)	216 (5.33)	247 (5.43)	953 (5.08)
Non-sedative antidepressants	140 (5.13)	229 (6.35)	230 (6.03)	227 (5.60)	262 (5.76)	1,088 (5.80)
Antipsychotics	56 (2.05)	102 (2.83)	118 (3.09)	125 (3.08)	111 (2.44)	512 (2.73)
MRAs	0 (0)	0 (0)	0 (0)	1 (0.02)	2 (0.04)	3 (0.02)
Other sedative drugs	25 (0.92)	18 (0.50)	54 (1.41)	56 (1.38)	70 (1.53)	223 (1.19)

BZDs, benzodiazepines; nBZRAs, non-benzodiazepine receptor agonists; MRAs, melatonin receptor agonists. *The incidence of nBZRA usage significantly increased between 2015 and 2019 (P < 0.05). **The incidence of BZD usage significantly decreased between 2015 and 2019 (P < 0.01). ^#^The incidence of antidepressant usage significantly increased between 2015 and 2019 (P < 0.05).

### Proportion and DDDs of Each Drug

Tables 3–5 show the number of prescribed outpatients, proportion, total doses, and DDDs for each drug. The most frequently prescribed drug for insomnia was estazolam (38.37%). However, the DDDs of zolpidem were higher than those of estazolam. The most commonly prescribed antidepressants, antipsychotics, and other sedative drugs were trazodone, olanzapine, and gabapentin, respectively. The incidence of trazodone use increased significantly each year from 2.85% in 2015 to 4.63% in 2019 (*P* < 0.05), whereas the percentage of mirtazapine decreased significantly from 1.89% in 2015 to 0.95% in 2019 (*P* < 0.05).

**Table 3:** Situation of prescribed benzodiazepine receptor agonists in outpatients with insomnia.

**Drug class/drug**	**Number of visits (%)**	**Total doses (mg)**	**DDDs**
**BZDs**			
Estazolam	6,190 (38.37)	169,086	56,362
Alprazolam	2,804 (17.38)	34,722	34,722
Clonazepam	680 (4.22)	33,112	4,139
Lorazepam	238 (1.48)	3,227	1,290.8
Diazepam	102 (0.63)	3,852.5	385.25
Nitrazepam	62 (0.38)	6,860	1,374
Oxazepam	36 (0.22)	12,900	259
Midazolam	14 (0.09)	2,400	160
**nBZRAs**			
Zolpidem	5,395 (33.44)	687,100	68,710
Zopiclone	252 (1.56)	30,510	4,068
Dexzopiclone	232 (1.44)	12,138	4,046

*BZDs, benzodiazepines; nBZRAs, non-benzodiazepine receptor agonists; DDDs, defined daily doses*.

**Table 4:** Situation of prescribed antidepressants in outpatients with insomnia.

**Drug class/drug**	**Number of visits (%)**	**Total doses (mg)**	**DDDs**
**Sedative antidepressants**			
Trazodone*	580 (3.60)	902,000	3,007
Mirtazapine#	202 (1.25)	129,600	4,320
Amitriptyline	146 (0.91)	158,000	2,107
Doxepin	23 (0.14)	38,500	385
**Non-sedative antidepressants**			
Sertraline	338 (2.10)	618,100	12,362
Citalopram	234 (1.45)	143,360	7,168
Escitalopram	139 (0.86)	46,340	4,634
Duloxetine	142 (0.88)	183,750	3,062.5
Venlafaxine	82 (0.51)	231,700	2,317
Paroxetine	78 (0.48)	53,320	2,666
Fluoxetine	42 (0.26)	42,460	2,123
Fluvoxamine	33 (0.20)	142,500	1,425

DDDs, defined daily doses. *The incidence of trazodone usage significantly increased between 2015 and 2019 (P < 0.05). ^#^The incidence of mirazapine usage significantly decreased between 2015 and 2019 (P < 0.05).

**Table 5:** Situation of prescribed antipsychotics, MRAs and other sedative drugs in outpatients with insomnia.

**Drug class/drug**	**Number of visits (%)**	**Total doses (mg)**	**DDDs**
**Antipsychotics**			
Olanzapine	303 (1.88)	27,820	2,782
Quetiapine	196 (1.21)	617,000	1,542.5
Clozapine	7 (0.04)	17,250	57.5
**MRAs**			
Agomelatine	3 (0.02)	1,400	56
**Other sedative drugs**			
Gabapentin	189 (1.18)	2,514,600	1,397
Pregabalin	27 (0.17)	66,000	220
Phenobarbital	7 (0.04)	11,880	118.8

*MRAs, melatonin receptor agonists*.

### Drug Combination

In total, 13.97% of outpatients with insomnia were prescribed two or more types of drugs for insomnia, with proportions of double, triple, and quadruple or more medications of 11.95, 1.77, and 0.24%, respectively (Table 6). Table 7 shows the detailed types of anti-insomnia drug combinations and their proportions in double medication combinations. Of the double medication combinations, 46.92% were benzodiazepine receptor agonists (BZRAs) combinations and 36.15% were BZRAs in combination with antidepressants. However, nearly half of the drugs used in combination with one another had similar pharmacological mechanisms that may have increased the risk of adverse reactions, including BZDs combined with nBZRAs (36.47%), BZDs combined with other BZDs (3.46%), and two BZRAs combined with antidepressants or antipsychotics (6.21%) (Supplementary Table 1).

**Table 6:** Trends of multidrug combinations prescribed in sampled outpatient insomniacs from 2015 to 2019.

	**Number of patients (%)**					
	**2015**	**2016**	**2017**	**2018**	**2019**	**Total**
Outpatient visits	2,385	3,098	3,252	3,485	3,919	16,133
Single drug	2,085 (87.42)	2,668 (86.12)	2,791 (85.82)	2,975 (85.37)	3,360 (85.86)	13,879 (86.03)
Multidrug combinations	300 (12.58)	430 (13.88)	461 (14.18)	510 (14.63)	553 (14.14)	2,254 (13.97)
Double combinations	260 (10.90)	366 (11.81)	381 (11.72)	453 (13.00)	470 (12.01)	1,930 (11.95)
Triple combinations	39 (1.63)	55 (1.78)	65 (2.00)	53 (1.52)	73 (1.87)	285 (1.77)
Quadruple or more combinations	1 (0.04)	9 (0.29)	15 (0.46)	4 (0.11)	10 (0.26)	39 (0.24)

**Table 7:** Trends of double medication combinations prescribed in sampled outpatients with insomnia between 2015 and 2019.

	**Number of patients (%)**					
	**2015**	**2016**	**2017**	**2018**	**2019**	**Total**
Outpatient visits	260	366	381	453	470	1,930
BZRAs combination	122 (46.92)	186 (50.82)	173 (45.41)	226 (49.89)	215 (45.74)	922 (47.77)
BZD + nBZRA	111 (42.69)	155 (42.35)	144 (37.80)	210 (46.36)	202 (42.98)	822 (42.59)
BZRA + antidepressant	94 (36.15)	115 (31.42)	129 (33.86)	144 (31.78)	153 (32.56)	635 (32.90)
BZD + antidepressant	68 (26.15)	74 (20.22)	84 (22.05)	86 (18.98)	96 (20.43)	408 (21.14)
nBZRA + antidepressant	26 (10.00)	41 (11.20)	45 (11.81)	58 (12.80)	57 (12.13)	227 (11.76)
BZRA + Antipsychotic	11 (4.23)	18 (4.92)	21 (5.51)	28 (6.18)	28 (5.96)	106 (5.49)
BZRA + antiepileptic	9 (3.46)	6 (1.64)	18 (4.72)	15 (3.31)	28 (5.96)	76 (3.94)
Antidepressants combination	10 (3.85)	12 (3.28)	12 (3.15)	10 (2.21)	20 (4.26)	64 (3.32)
Antidepressant + antipsychotic	12 (4.62)	29 (7.92)	28 (7.35)	22 (4.86)	12 (2.55)	103 (5.34)
Other combinations	2 (0.77)	0 (0)	12 (3.15)	8 (1.77)	14 (2.98)	36 (1.87)

*BZD, benzodiazepine; BZRAs, benzodiazepine receptor agonists; nBZRA, non-benzodiazepine receptor agonist*.

## Discussion

In the present study, we described the demographic characteristics of patients prescribed medications for insomnia and investigated drug trends using prescription data between 2015 and 2019 in a province in China. The number of outpatients prescribed medications for insomnia increased throughout the study period, and the mean age of those patients decreased. BZDs were the medications most often used to treat insomnia. However, a decline in the incidence of BZD usage and increases in nBZRA and antidepressant usage were observed.

The incidences of insomnia and associated prescriptions have increased worldwide over the past two decades (14, 19–22), and we found a similar yearly trend in visits by patients prescribed medications for insomnia in this study. Previous studies have found that age is a risk factor for insomnia, and the prevalence of insomnia increases with age (3, 14). Although patients with insomnia aged 45–65 years were prescribed most of the anti-insomnia drugs in this study, the proportion of patients over 65 years of age was 43.54%, which was similar to that in Begum's study which was conducted in Australia (12). However, the mean age of patients with insomnia who were prescribed medication was younger. These results indicate that patients who need pharmacological treatment to control insomnia symptoms are becoming younger. Women are reportedly at an increased risk of insomnia, with a 1.4–1.5 times higher risk ratio than men (23, 24). The present study also found that there were nearly 1.42 times more women with insomnia than men, and female patients tended to be younger. This may be due to hormonal changes, socially sanctioned culture, comorbidity, and the influence of psychological factors on women (24). Anti-insomnia prescriptions were also most frequently prescribed in the departments of cardiology and neurology. One possible reason of this finding is that insomnia is a common complication of cardiovascular and cerebrovascular diseases, and it has been proved that insomnia is a risk factor for the occurrence and death of these diseases (25, 26), hence, doctors in these two departments pay more attention to the treatment of insomnia. On the other hand, traditional Chinese culture may also play a role, as stigma may be attached to psychiatric disorders for Chinese; thus, patients are unwilling to seek treatment in the department of psychiatry.

The prescription of BZDs has reportedly increased in some countries, such as the USA (27), Korea (14), and Sweden (28) but declined in Canada (29) and Spain (30). However, the proportion of BZD prescriptions for insomnia has decreased and the proportion of nBZRA prescriptions has increased in the USA (11) and Australia (12). We found a similar trend in BZD and nBZRA prescriptions for insomnia in China between 2015 and 2019. BZDs have been widely reported to have serious adverse effects (9, 10), and guidelines in China recommend nBZRAs as the preferred medicine for insomnia (8, 17). Nevertheless, BZDs were still the most commonly used type of medicine for insomnia in the present study, with similar results reported in Japan (13), Korea (14), and Beijing in China (31). This serves to remind us to pay attention to the rational use of BZDs. The most frequently prescribed nBZRA for insomnia in most countries is zolpidem (12–14), including China in the current study. However, there are differences among countries regarding BZDs. For example, the most frequently prescribed BZD for insomnia is temazepam in Australia (12), brotizolam in Japan (13), lorazepam in Korea (14), and estazolam in China in the present study and Zhong's study (31). This difference may be due to the variety of BZDs and different health insurance policies among countries.

Sedative antidepressants can be used to treat insomnia in patients with comorbid depression or cases of first-line pharmacological treatment failure (6, 9). Nearly 10% of patients with insomnia in the USA are prescribed sedative antidepressants (22), with trazodone being the most common (20). In contrast, mirtazapine and amitriptyline are more commonly prescribed sedative antidepressants for patients with insomnia in Australia, with annual increases of 4.3 and 5.1%, respectively (12). Similar results were found in Finland between 2000 and 2010, which were analyzed according to DDD per 1,000 inhabitants per day (32). Approximately, 5.08% of the prescriptions for insomnia in our study were sedative antidepressants, which is similar to reported proportion of sedative antidepressants (6.82%) in Beijing in China (31), and an increase in the incidence of whole antidepressant usage, but not that of sedative antidepressants, was observed. We also found an increase in the proportion of trazodone prescriptions, but a reduction in the proportion of mirtazapine prescriptions. Interestingly, the most potentially used sedative antidepressant was trazodone, which was analyzed according to prescription proportion, whereas mirtazapine was analyzed based on DDDs. This result was due to the daily dose difference between mirtazapine and trazodone. Trazodone was prescribed at 25–100 mg daily in our data, whereas mirtazapine was prescribed more frequently at 30–45 mg daily, which was larger than the dose required to treat insomnia (8). Moreover, non-sedative antidepressants, such as SSRIs and serotonin/norepinephrine reuptake inhibitors, are not recommended for insomnia in American and European guidelines (2, 7), but they can be used in combination with BZRAs in patients with comorbid depression in China (8, 17). Although SSRIs and serotonin/norepinephrine reuptake inhibitors reportedly induce insomnia (33, 34), randomized controlled trials have shown that nBZRAs co-administered with SSRIs significantly improve total sleep time, sleep onset latency, and sleep quality in patients with depressive disorder (35–37). Sertraline was the most frequently prescribed non-sedative antidepressant in patients with comorbid depression and insomnia.

Evidence for antipsychotics as a treatment for insomnia is weak, and they are not routinely used to treat insomnia unless the patient has a psychiatric disorder (17). Quetiapine is the most commonly used antipsychotic for insomnia in many countries, and its prescription proportion for insomnia is nearly 11% in the USA, but <4% in Australia and Korea (14, 20, 32, 38). However, in this study, we found that the most commonly used antipsychotic for insomnia in China was olanzapine, and only 1.21% of prescriptions were quetiapine. Gabapentin and pregabalin are often used to treat insomnia with comorbid chronic pain conditions or epilepsy; however, their efficacy and safety have not been evaluated systematically to date (6, 8, 39). In the present study, we found that 1.18% of patients with insomnia were prescribed gabapentin and 0.17% were prescribed pregabalin. New types of the anti-insomnia drugs MRAs and ORAs, such as suvorexant, agomelatine, and ramelteon, are effective against insomnia and reduce sleep latency and improve sleep efficiency and quality (40–42). Only three patients with insomnia were prescribed agomelatine, and no patient was prescribed suvorexant or ramelteon. Agomelatine was approved by the China Food and Drug Administration in 2015 and was not included in the national health insurance until 2019, whereas ramelteon and suvorexant have not yet been approved in China.

Multidrug combinations are not routinely recommended for insomnia unless patients experience multiple monotherapy failures or comorbidities with psychiatric disorders (8, 17). Combination treatment with low doses of drugs with different pharmacological effects could achieve some benefits from each drug class while minimizing adverse effects (35, 36). However, combining drugs with similar or identical mechanisms of action may result in an increased risk of adverse effects. Kaufmann et al. found that half of the patients visiting the emergency department who experienced BZD-related adverse events were taking a combination of BZDs and nBZRAs and were at an almost four times greater risk of serious disease (43). According to the National Ambulatory Medical Care Survey in the United States, the co-prescription rate of BZDs and nBZRAs in all outpatient prescriptions increased notably from 0% in 1993 to 0.4% in 2010 (11), as did the co-prescription rate of BZDs with other sedating medications between 2003 and 2015 (44). We found that 13.97% of prescriptions for insomnia were multidrug combinations, and their number increased annually. Double medication combinations were the most common, and nearly half of the double medication combinations were combinations with BZRAs. Worryingly, nearly half of the drug combinations, such as BZDs + nBZRAs, double BZDs, and double nBZRAs, had similar pharmacological mechanisms, which may lead to an increased risk of serious adverse effects. These results serve as a reminder to be vigilant of unreasonable drug combinations when treating insomnia.

Nevertheless, this study had some limitations. Prescription data were obtained from six hospitals in Zhejiang province that participated in the Hospital Prescription Analysis Cooperative Project, which may have resulted in sampling bias that these data may not accurately represent the Zhejiang province or the whole of China. We analyzed prescription information for outpatient insomnia but could not confirm whether patients took their medications as prescribed. Our data were also obtained using random sampling; hence, some information of drug combinations might have been omitted, and evaluating the continuous medication changes in patients with insomnia was difficult. Future studies will need to evaluate real-world changes in anti-insomnia medications by continuous follow-up of patients with insomnia. The prescription data of patients with insomnia in different regions of China are also necessary to be collected to analyze the situation of pharmacological treatments for insomnia in the whole of China.

## Conclusion

The number of outpatients with insomnia receiving pharmacological treatment increased yearly, whereas their age tended to decrease. BZDs are still the most commonly prescribed medications for insomnia, but the prescription rates of nBZRAs and antidepressants are increasing. It should be noted that the number of patients with insomnia who were administered combination drugs increased yearly, and nearly half of them were prescribed drugs with similar pharmacological mechanisms. This may lead to an increased risk of serious adverse effects. These results will serve as a reference for more robust monitoring and rational use of pharmacological treatments for insomnia.

## Data Availability Statement

The original contributions presented in the study are included in the article/Supplementary Material, further inquiries can be directed to the corresponding author.

## Ethics Statement

The studies involving human participants were reviewed and approved by the Ethics Committee of Sir Run Run Shaw Hospital, School of Medicine, Zhejiang University (reference number: 20210924-33). Written informed consent from the participants' legal guardian/next of kin was not required to participate in this study in accordance with the national legislation and the institutional requirements.

## Author Contributions

GL and LZ: conceptualization and data curation. GL, YZ, and LC: formal analysis. GL, ZY, and YZ: investigation. ZY: methodology. GL: writing—original draft. YZ, LC, and LZ: writing—review and editing. LZ: supervision. All authors have read and agreed to the published version.

## Funding

This work was supported by the Medicine and Health Science and Technology plan projects of Zhejiang Province (2019333731) and the Scientific Research Projects of Hospital Pharmacy of Zhejiang Pharmaceutical Association (2014ZYY07).

## Conflict of Interest

The authors declare that the research was conducted in the absence of any commercial or financial relationships that could be construed as a potential conflict of interest.

## Publisher's Note

All claims expressed in this article are solely those of the authors and do not necessarily represent those of their affiliated organizations, or those of the publisher, the editors and the reviewers. Any product that may be evaluated in this article, or claim that may be made by its manufacturer, is not guaranteed or endorsed by the publisher.
