# Supplementary material for: Trends in Prescriptions for Insomnia in a Province in China Between 2015 and 2019
Source: Front Psychiatry. 2022 Jun 20;13:915823. doi: 10.3389/fpsyt.2022.915823 (PMC9251053; doi:10.3389/fpsyt.2022.915823)
Supplement: Supplementary file 1 [file Table_1.DOCX]

Supplementary Table 1. Combination of drugs with similar pharmacological mechanisms in outpatients with insomnia.

|  | Number of outpatients | Proportion in multidrug combinations（%） |
| --- | --- | --- |
| Double combinations | 951 | 40.60 |
| BZD + nBZRA | 822 | 36.47 |
| BZD + BZD | 78 | 3.46 |
| nBZRA + nBZRA | 10 | 0.44 |
| SNRI + SSRI | 1 | 0.04 |
| Other combination | 4 | 0.18 |
| Triple combinations | 168 | 7.45 |
| 2 nBRZAs + Antidepressant | 121 | 5.37 |
| 2 BRZAs + Antipsychotic | 19 | 0.84 |
| 3 BRZAs | 20 | 0.89 |
| Other combination | 8 | 0.35 |
| Quadruple or more combinations | 35 | 1.55 |

BZD, benzodiazepines; nBZRA, non-benzodiazepine receptor agonist; SSRI, selective serotonin reuptake inhibitor; SNRI: serotonin/norepinephrine reuptake inhibitors.
